# Accuracy of genomic selection for alfalfa biomass yield in different reference populations

**DOI:** 10.1186/s12864-015-2212-y

**Published:** 2015-12-01

**Authors:** Paolo Annicchiarico, Nelson Nazzicari, Xuehui Li, Yanling Wei, Luciano Pecetti, E. Charles Brummer

**Affiliations:** Council for Agricultural Research and Economics (CREA), Research Centre for Fodder Crops and Dairy Productions, 29 viale Piacenza, 26900 Lodi, Italy; Department of Plant Sciences, North Dakota State University, 1340 Administration Avenue, Fargo, ND 58108 USA; Plant Sciences Department, University of California, Davis, Plant Breeding Center, One Shields Avenue, Davis, CA 95616 USA

**Keywords:** Alfalfa, Breeding strategy, Genomic selection, Genotyping-by-sequencing, Lucerne, *Medicago sativa*, Missing data imputation, Yield

## Abstract

**Background:**

Genomic selection based on genotyping-by-sequencing (GBS) data could accelerate alfalfa yield gains, if it displayed moderate ability to predict parent breeding values. Its interest would be enhanced by predicting ability also for germplasm/reference populations other than those for which it was defined. Predicting accuracy may be influenced by statistical models, SNP calling procedures and missing data imputation strategies.

**Results:**

Landrace and variety material from two genetically-contrasting reference populations, i.e., 124 elite genotypes adapted to the Po Valley (sub-continental climate; PV population) and 154 genotypes adapted to Mediterranean-climate environments (Me population), were genotyped by GBS and phenotyped in separate environments for dry matter yield of their dense-planted half-sib progenies. Both populations showed no sub-population genetic structure. Predictive accuracy was higher by joint rather than separate SNP calling for the two data sets, and using random forest imputation of missing data. Highest accuracy was obtained using Support Vector Regression (SVR) for PV, and Ridge Regression BLUP and SVR for Me germplasm. Bayesian methods (Bayes A, Bayes B and Bayesian Lasso) tended to be less accurate. Random Forest Regression was the least accurate model. Accuracy attained about 0.35 for Me in the range of 0.30-0.50 missing data, and 0.32 for PV at 0.50 missing data, using at least 10,000 SNP markers. Cross-population predictions based on a smaller subset of common SNPs implied a relative loss of accuracy of about 25 % for Me and 30 % for PV. Genome-wide association analyses based on large subsets of *M. truncatula*-aligned markers revealed many SNPs with modest association with yield, and some genome areas hosting putative QTLs. A comparison of genomic vs. conventional selection for parent breeding value assuming 1-year vs. 5-year selection cycles, respectively, indicated over three-fold greater predicted yield gain per unit time for genomic selection.

**Conclusions:**

Genomic selection for alfalfa yield is promising, based on its moderate prediction accuracy, moderate value of cross-population predictions, and lack of sub-population structure. There is limited scope for searching individual QTLs with overwhelming effect on yield. Some of our results can contribute to better design of genomic selection experiments for alfalfa and other crops with similar mating systems.

**Electronic supplementary material:**

The online version of this article (doi:10.1186/s12864-015-2212-y) contains supplementary material, which is available to authorized users.

## Background

Crop yield, which generally is the main objective of breeding programs, has been improved essentially by phenotypic selection, owing to inability of marker development to ensure sufficient genome coverage for this complex trait. This holds true also for alfalfa (*Medicago sativa* L. subsp. *sativa*), which is the most grown perennial forage legume globally [1] with potential interest also as a dual-purpose crop for bioenergy and protein feed [2]. Yield breeding progress for this crop has been particularly slow compared with other major field crops, owing to low breeding investment, long selection cycles, high material evaluation cost, impossibility to capitalize on harvest index, low narrow-sense heritability (*h*_*N*_^*2*^) partly due to a large component of non-additive genetic variance, outbreeding mating system associated with severe inbreeding depression, and high genotype-environment interaction [3, 4]. Published estimates of *h*_*N*_^*2*^ for alfalfa biomass yield ranged from 0.15 to 0.30, including the value of 0.21 observed for one set of Italian alfalfa genotypes that was also used for the current study [1]. Such low *h*_*N*_^*2*^ values, and the long and expensive selection cycles, emphasize the practical importance of exploring selection procedures for higher biomass yield that use marker information as a partial substitute for field-based selection [5].

Early research work aimed to identify molecular markers strongly linked to quantitative trait loci (QTL) for alfalfa forage yield could rely on about 150–200 RFLP, AFLP, SSR or RAPD markers [6–9]. Of necessity with so few markers, QTL discovery focused on a limited genetic base represented by F1 progenies of a bi-parental population, which, along with the expected absence of individual markers with high yield effect, limited the practicality of a marker-assisted selection program. The availability of large numbers of SNP which could be turned into markers [10, 11] has enhanced the opportunities for marker-assisted selection, allowing for exploring wider genetic bases through association mapping [12, 13]. The development of an alfalfa Illumina Infinium SNP array containing about 10,000 SNP markers has provided a high-throughput platform [14]. Such high marker number may also allow for sufficient genome saturation for genomic selection, by which phenotyping and genotyping data of a genotype sample representing a target genetic base (reference population) are combined into a model that estimates breeding values for future plant selection [15, 16]. Simulation and empirical studies proved that genomic selection is superior to conventional marker-assisted selection based on limited marker numbers in prediction of breeding values for complex polygenic traits, such as crop yield [17, 18].

The recent development of methods to genotype directly from sequence data, such as genotyping-by-sequencing (GBS) [19], can decrease the cost of marker-based selection for production traits compared to SNP array platforms. A GBS-based high-density linkage map for tetraploid alfalfa including over 3500 SNP markers has been constructed [20]. However, GBS commonly generates large amounts of missing data that must be imputed before fitting a genomic prediction model. Imputation method [21] and the genomic selection model [16] may influence prediction accuracy, i.e., the correlation between predicted and true breeding values.

Genomic prediction accuracy and narrow-sense heritability of the yield trait are crucial genetic parameters for the comparison of selection strategies in terms of expected yield gain, for the popular scheme of parent selection based on half-sib progeny responses [4]. Genomic selection models with accuracies as high as 0.66 for cross-validation within a given location and cycle and 0.40 for predicting genotype yields in a following cycle were obtained for parent material phenotyped and selected as individual cloned plant [22]. However, the ideal phenotypic data on which to base a genomic selection model for perennial forage crops would be sward plot yield of half-sib progenies rather than cloned space-planted parents, to closely represent actual production environments and to focus on additive genetic variance, the relevant variance for synthetic variety breeding [4]. Thus, successful application of genomic prediction models to a half-sib breeding program would provide better evidence that GS could accelerate yield gain in alfalfa. Also, understanding how well genomic selection models can predict yield in germplasm/reference populations other than those for which they were defined would help clarify the cost of incorporating GS models into a breeding program [4].

This study provides an unprecedented, thorough assessment of the potential value of genomic selection for assessing alfalfa parent breeding values for biomass yield based on GBS data. Results are provided for two reference populations that represent quite distinct genetic bases, namely, one assembled from elite landrace and variety germplasm adapted to the sub-continental climate conditions of Northern Italy [23], and the other constituted by repeated intercrossing of genotypes from three populations that were top-performing across Mediterranean-climate environments of the Western Mediterranean basin [24]. These populations differed also for conditions of biomass yield phenotyping. Genomic selection models for parent selection were constructed from phenotypic data of their dense-planted half-sib progenies, assessing their selection accuracy for different SNP calling procedures, strategies and algorithms for missing data imputation, and prediction models. In addition, we performed a genome-wide association analysis for a subset of *M. truncatula*-aligned SNP markers, and verified the cross-population accuracy of the genomic selection models.

## Results

### Phenotypic variation

Half-sib progenies differed for total dry matter (DM) yield in both populations (*P* < 0.001). Best linear unbiased predictors (BLUP) values ranged from 19.8 to 28.1 t/ha for the 124 progenies of parent genotypes from the reference population PV originated in the Po Valley, and from 6.4 to 8.8 t/ha for the 154 progenies of parent material from the population Me adapted to Mediterranean-climate environments. The difference in yield levels between populations reflected the different duration of their respective phenotyping experiments (3 years for PV vs. 1 year for Me). In both populations, the distribution of parent breeding values (as inferred from yield values of their half-sib progenies) visually approached the expected normal distribution [see Additional file 1: Figure S1].

### GBS data

**Fig. 1:**
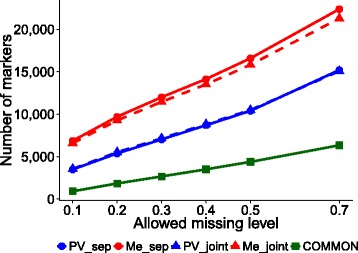
Number of SNP markers for different genotype missing data thresholds and SNP calling strategies. Results for 124 genotypes of the Po Valley population (PV) and 154 genotypes of the Mediterranean population (Me) subjected to separate SNP calling (data sets PV_Sep and Me_Sep), or joint SNP calling with subsequent application of missing data thresholds to separate populations (PV_Joint and Me_Joint) or joint populations (COMMON)

The total number of polymorphic SNPs without regard to the amount of missing data after applying read-depth filtering amounted to 68,972 for PV and 77,610 for Me. Obviously, increasingly stricter thresholds for the number of genotypes with missing data resulted in progressively fewer SNPs available for genomic selection (Fig. 1). SNP number, however, remained relatively high even at a fairly stringent missing data thresholds, e.g., over 11,000 for the population Me and 7000 for PV at the 0.20 threshold. In general, marker counts for population PV were lower than those for Me, probably reflecting the more selective adapter used for GBS library construction of this population. Marker counts for each population were very similar between separate and joint SNP calling, with just a slight advantage in marker number for separate SNP calling in the Me population (Fig. 1).

### Population structure

**Fig. 2:**
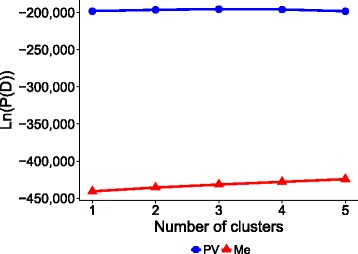
STRUCTURE analysis of sub-populations. Log likelihood values of posterior probability as a function of the number of sub-populations, separately for the Po Valley (PV) and the Mediterranean (Me) populations

In both populations, the substantially flat response of the log likelihood values of posterior probability for increasing numbers of possible sub-populations indicated the absence of population structure (Fig. 2). This result was confirmed by results of Evanno’s criterion, as well as by the lack of genotype groups with consistently greater genetic similarity in the kinship matrix analysis [see Additional file 2: Figure S2]. These findings supported the omission of a parameter for population structure in genomic selection models of both populations.

### SNP calling procedures and imputation method in genomic selection models

**Fig. 3:**
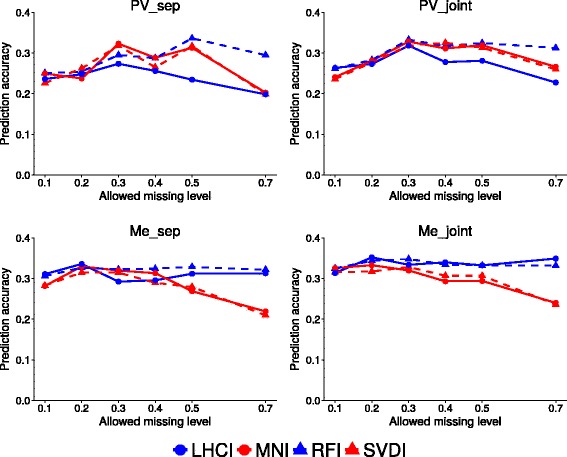
Prediction accuracy for different genotype missing data imputation methods, SNP calling strategies and missing data thresholds. Results for four imputation methods (MNI, Mean imputation; SVDI, Singular value decomposition imputation; RFI, Random forest imputation; LHCI, Localized haplotype clustering imputation) applied to Po Valley (PV) and Mediterranean (Me) data sets subjected to separate SNP calling (PV_Sep and Me_Sep) or joint SNP calling (PV_Joint and Me_Joint), using Support Vector Regression with linear kernel

SNP calling for PV and Me was envisaged either separately (data sets PV_Sep and Me_Sep), or jointly followed by application of missing data thresholds to separate populations (data sets PV_Joint and Me_Joint). SNP calling procedures were assessed with reference to Support Vector Regression with linear kernel (SVR-lin) and Ridge Regression BLUP, since these models displayed higher predictive accuracies than other genomic selection models in following analyses. Prediction accuracy values were obtained for the two data sets and a combination of four imputation methods, namely, Mean imputation (MNI), Singular value decomposition imputation (SVDI), Random forest imputation (RFI) and Localized haplotype clustering imputation (LHCI). The results highlighted the merit of RFI for both data sets, both using SVR-lin (Fig. 3) and Ridge Regression BLUP [see Additional file 3: Figure S3]. This method performed slightly better than, or comparably to, any other method, with the exception of the data set PV_Sep for the missing data thresholds 0.20 and 0.30 (Fig. 3). As expected, the differences in accuracy between imputation algorithms increased with relaxed thresholds for missing genotype data (implying greater amounts of estimated missing data) (Fig. 3).

Results in Fig. 3 also revealed the trend towards greater accuracy of data sets of the two populations that underwent joint SNP calling (PV_Joint, Me_Joint), compared with data sets subjected to separate SNP calling (PV_Sep, Me_Sep). Averaged across the six missing data thresholds and RFI, the accuracy gain obtained by joint SNP calling was 10.1 and 6.4 % for PV and Me populations, respectively.

In general, the prediction accuracy of parent breeding values was higher for Me germplasm than PV material. With reference to the preferable configuration of joint SNP calling and RFI using the SVR-lin model, the accuracy response as a function of genotype missing data thresholds was roughly flat for the Me population (with values around 0.35 in the range of 0.30–0.50 missing data), while showing a peak of 0.32 accuracy at 0.50 missing data for PV germplasm (Fig. 3). Such top-performing genomic selection models included at least 10,000 SNP markers (Fig. 1). The list of detected SNPs and their identifying flanking sequences are provided in [Additional file 4] and [Additional file 5].

### Comparison of genomic selection models

**Fig. 4:**
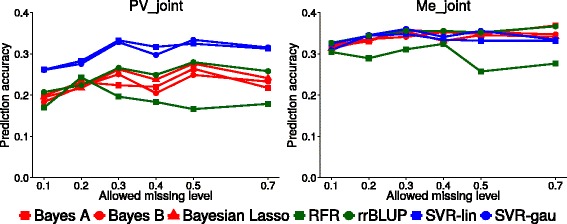
Prediction accuracy of four genomic selection models at different genotype missing data thresholds. Results for Support Vector Regression with linear (SVR-lin) and gaussian (SVR-gau) kernel, Random Forest Regression (RFR), Ridge Regression BLUP (rrBLUP), Bayes A, Bayes B and Bayesian Lasso models applied to Po Valley (PV_Joint) and Mediterranean (Me_Joint) data sets subjected to joint SNP (random forest imputation of missing data)

Support Vector Regression using Linear and Gaussian Kernel, Ridge regression BLUP, Random Forest Regression and three Bayesian models, namely, Bayes A, Bayes B and Bayesian Lasso, were compared in terms of predictive accuracy for the preferable configuration of joint SNP calling and RFI. We found only limited differences between methods on Me germplasm (Fig. 4), where only Random Forest Regression stood out as the worst-performing candidate. On PV germplasm, however, Support Vector models outperformed all other models, with a constant advantage of about 0.05–0.07 on the third best-performing model. In general, the two Support Vector kernels performed comparably and with no clear discernible trend in accuracy. However, SVR-lin resulted in shorter computation times.

Among Bayesian methods, Bayesian Lasso tended towards greater accuracy than Bayes A and B for PV material, whereas the three methods performed comparably for Me germplasm (Fig. 4). On average, Ridge Regression BLUP slightly outperformed Bayesian methods, a trend confirmed also in data sets that underwent separate SNP calling (PV_Sep and Me_Sep) (data not reported).

### Genomic selection: cross-population predictions

This assessment was carried out using the COMMON data set, which included only the SNPs that satisfied filtering criteria simultaneously for PV_Joint and Me_Joint data sets. This data set exhibited relatively small SNP numbers (Fig. 1), which averaged 37 % of those featuring the smaller of the joint data sets (PV_Joint) across genotype missing data thresholds.

**Fig. 5:**
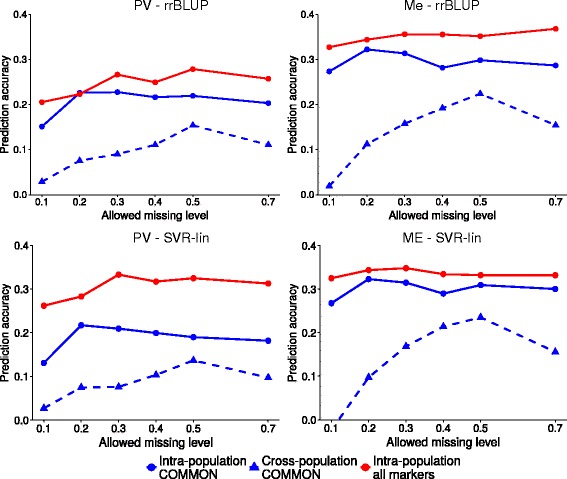
Accuracy of genomic selection for intra-population and cross-population prediction strategies at different genotype missing data thresholds. Intra-population prediction using all markers subjected to joint SNP calling (PV_Joint and Me_Joint data sets) or only markers satisfying the common filtering criteria (COMMON data set), and cross-population predictions using the COMMON data set, for Po Valley (PV) and Mediterranean (Me) populations, using Support Vector Regression with linear kernel or Ridge Regression BLUP (random forest imputation of missing data)

The accuracies of cross-population predictions by SVR-lin and Ridge Regression BLUP models were compared with intra-population predictions by the same models, using same markers (COMMON data set). Fig. 5 also includes, as a reference, intra-population prediction accuracies based on all available markers (PV_Joint and Me_Joint data sets). The advantage of using the complete marker data set was high for PV germplasm and only marginal for the Me one, especially considering the much higher number of SNPs available.

In both populations, cross-population prediction accuracies were definitely lower than intra-population ones based on same markers (Fig. 5). However, the relative disadvantage of cross-population prediction decreased for more relaxed thresholds of genotype missing data and reached a minimum at 50 % missing data, where the relative loss of accuracy was comparable for both models. This loss amounted to about 28 and 25 % for PV and Me germplasm, respectively, using SVR-lin, and 30 and 25 % for PV and Me germplasm, respectively, using Ridge Regression BLUP.

### Genome-wide association analysis

We selected for *M. truncatula* alignment the SNP markers of PV_Joint and Me_Joint data sets that tended to maximize intra-population prediction accuracy, namely, those of 50 % missing data threshold for PV and 30 % threshold for Me (both imputed with RFI). Non-aligned markers (placed on the fictitious chromosome N) were 28.1 % for PV and 24.5 % for Me populations. The aligned markers were 7544 for PV and 8648 for Me populations, resulting in an average physical distance between SNPs of 40 Kbp for PV and 34.5 Kbp for Me.

**Fig. 6:**
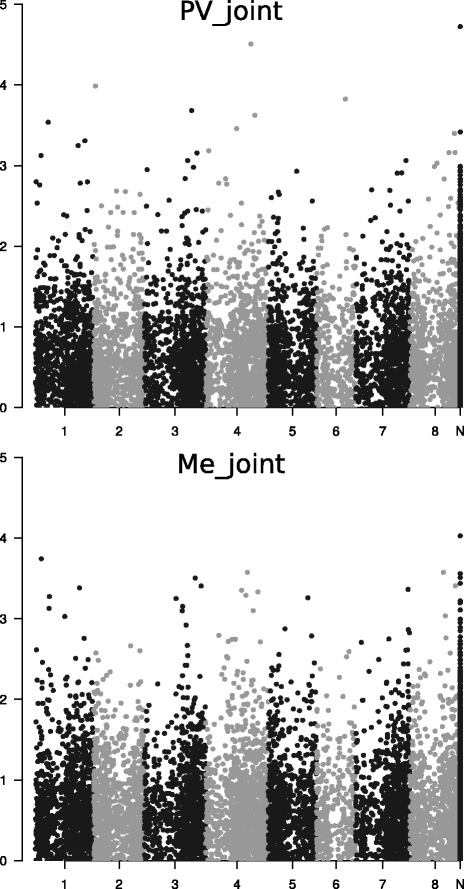
Association (Manhattan plot) of *M. truncatula*-aligned SNP markers with total dry matter yield. Results for Po Valley (PV) and Mediterranean (Me) populations

As expected for a complex traits such as crop yield, we found a high number of SNPs that tended towards a modest association with the trait (Fig. 6). The simultaneous inspection of the Manhattan plots for the two populations suggested some consistency of genome areas hosting putative QTLs, such as those around the end of the chromosomes 1 and 6, or an area in the last third of chromosome 8 (Fig. 6).

## Discussion

The phenotyping of PV material, which extended over 3 years, was consistent with the actual alfalfa cycle duration in Northern Italy. Parent breeding values based on 3-year DM yield were the result of intrinsic yielding ability as expressed by short-term DM yield, and persistence. The latter trait may depend on the plant’s ability to accumulate assimilates in the root for further regrowth under moisture-favorable conditions and to survive across stress periods by various physiological mechanisms under unfavorable conditions [25]. Both components of persistence were likely to be relevant under the moderate-drought stress phenotyping conditions that featured PV material. Hence, PV parent breeding value was based on a more complex and partly different DM yield trait relative to Me parent breeding value, which reflected only intrinsic yielding ability as revealed in the short term. The somewhat lower prediction accuracy observed for biomass yield of PV material relative to Me germplasm (about 0.32 vs. 0.35 for best-performing models) can be the result of greater complexity of its yield trait (as determined by persistence besides intrinsic yield potential), smaller genotype sample or lower number of SNPs that were available for this population.

SNP marker number made available by GBS in these data sets was in the range of 7000-11,000 for reasonably low rates of missing data. These values compare favorably with an earlier assessment of GBS in alfalfa [20], while approaching the SNP numbers obtainable by Illumina Infinium SNP array [14]. Compared with GBS, Infinium array has a higher cost per data point but also necessarily expensive genotyping experiments, owing to its need for large numbers of samples to be analyzed simultaneously. Our GBS-generated marker numbers might roughly suffice for genome exploration of broadly-based alfalfa populations, considering that at least 1000 SNP markers were estimated as necessary for a narrow-based population [13]. However, suboptimal SNP numbers might occur when attempting cross-population predictions, as we observed with the COMMON data set.

On average, the original GBS protocol by [19] as applied to Me material resulted in greater SNP number than the modified protocol applied to PV germplasm. Without ruling out the effect of genetic differences between populations, this result suggests that greater amplification of fewer target sites was not a useful strategy to limit the amount of missing data resulting from insufficient read depth. In contrast, this strategy showed some merit for soybean [26]. The consistent use of the same restriction enzyme limited the occurrence of distinct SNP markers for the two data sets. Indeed, the joint SNP calling in the two sets produced a sizable increase of genomic prediction accuracy, suggesting some advantage of the pooled information from the two data sets to improve the SNP calling quality for each data set. Joint SNP calling is operationally necessary to ensure the same SNP naming across different data sets in alfalfa and other crops that lack a stable reference genome and a public repository of unique SNP identifiers. The different GBS protocol, and genetic differences between populations, may account for the fact that the COMMON data set contained only a minority of the total SNP markers from Me_Joint and PV_Joint data sets, instead of approaching the SNP number of the more restrictive PV_Joint set.

Prediction accuracy was also affected by missing data imputation method, for which RFI emerged as a solid choice for the current unordered SNP data, as well as by the adopted statistical model for genomic selection. The effect of allowed missing data thresholds on prediction accuracy, which mostly displayed an accuracy peak in the range of 30–50 %, was consistent with the expected trade-off between increased information (more markers) and increased noise (higher imputation errors) arising from increasing thresholds.

The good performance of SVR models in this study agrees with the theoretical expectation of high accuracy for these methods when applied to traits that involve many QTLs with small individual effects [27, 28]. This was particularly true for PV germplasm, where genome-enabled predictions were more difficult than for Me because of various factors (greater complexity of the yield trait; less test genotypes; less markers), possibly because of the recognized value of SVR in high-noise conditions [29]. SVR and Ridge Regression BLUP performed were similarly for Me germplasm. The good performance of the latter method agrees with theoretical expectations [15, 16].

The complexity of the alfalfa biomass yield trait was confirmed by the high number of putative QTLs that emerged for both data sets from genome-wide association analysis. We believe that mapping individual QTLs for yield holds lower practical interest than genomic selection, for yield improvement programs of alfalfa and probably other crops.

The observed lack of sub-population structure facilitates the application of genomic selection by allowing for the adoption of simple genomic selection models. Lack of structure was purposefully searched for in the development of the Me, through repeated intercrossing of progenies generated by intercrossing genotypes from different populations. For population PV, whose genotype sample derived from several landraces and varieties from Northern Italy, lack of sub-population structure was not quite expected. A reason for this finding could be much higher intra-population variation relative to inter-population variation, which emerged for PV material from the study of several morphophysiological traits [23] and recent studies of SSR- and SNP-based genetic diversity (Annicchiarico et al. unpublished data). The population used by [22] in their prediction experiment was a strain cross of three cultivars subsequently intercrossed for two generations. It likewise had no sub-population structure. The pattern of these three very different populations suggests that application of marker prediction methods will not be limited within breeding programs by population structure.

The current predictions of parent breeding values proved much less accurate than predictions of alfalfa yield responses of cloned parents in an earlier study [22], likely because of the large extent of non-additive genetic variation reported consistently for this trait in various genetic studies [4] including one for the population PV [1]. Hence, focusing on parent breeding values (i.e., those of relevance in synthetic variety breeding) is of paramount importance for a realistic assessment of genomic selection in alfalfa and other crops with a similar mating system. It should be noted, however, that even a genomic prediction accuracy of breeding values (*r*_*A*_) around 0.32 (as achieved for PV material) can be promising for genomic selection. Considering the estimated narrow-sense heritability (*h*_*N*_^*2*^) of 0.21 reported for the same set of PV parent plants [1], selection cycles of 1 year for genomic selection (including genotyping plus polycrossing of selected genotypes) and 5 years for conventional parent selection based on half-sib progeny test (year 1, half-sib seed production from polycrossed replicated candidate parents; years 2–4, half-sib biomass yield evaluation; year 5, polycrossing of selected genotypes) and same selection intensity for both selection strategies, the comparison of genomic vs. conventional selection in terms of predicted yield gain per unit time reduces, according to formulae in [4], to *r*_*A*_ vs. (*h*_*N*_/5). This would indicate over three-fold greater efficiency for genomic selection [0.32 vs. (0.46/5)], assuming no degradation in the predictive power of the model across a few selection cycles (which may substantially hold, considering the fairly slow change in marker frequency expected for so high a number of loci subjected to selection). In addition, genomic selection is likely to allow for higher numbers of evaluated candidate parents (hence, higher selection intensity) than conventional selection, for same evaluation costs. GE interaction effects are bound to decrease genomic prediction accuracies [16] and yield gains over a target region, but this is applies as well to conventional selection, depending in all cases on the consistency of phenotyping or selection conditions with those prevailing in cropping environments [4]. Models developed across two locations actually had slightly higher accuracies for predicting yield of individual genotypes in a subsequent generation than models from either location independently [22], demonstrating that if extreme GE interactions are not present, broader inference across locations is desirable. For PV germplasm, the moderate summer drought stress conditions adopted for phenotyping and assessment of *r*_*A*_ and *h*_*N*_ parameters were suitable for minimizing GE interactions across Northern Italy [30]. Since a model’s prediction accuracy evaluated by cross-validations within the training population (as done here) may overestimate the model accuracy for selection within other genotypes of the same reference populations, future research work will compare genomic vs. phenotypic selection on the basis of actual yield gains obtained from selection within an independent genotype sample of the PV population. Other work will assess the potential of genomic selection for predicting breeding values of Me germplasm across a range of target environments, including some with severe drought or salinity stress.

That we observed only moderate loss of accuracy for cross-population predictions relative to within-population predictions is fairly surprising, especially considering the contrasting origin and the different duration and environmental conditions for biomass yield assessment of the two populations. Quite poor cross-population predictions were reported for wheat [31], in the presence of partly related populations evaluated under the same phenotyping conditions. The current result was probably favored by the high rate of within-population genetic variation that is typical of alfalfa, to which the development of broadly-based reference populations further contributed. It provides further support for the introduction of genomic selection in alfalfa breeding programs, whose selection is frequently carried out simultaneously on different germplasm pools.

## Conclusions

Our results indicate that genomic selection for alfalfa biomass yield is promising, based on its moderate prediction accuracy, moderate value of cross-population predictions, and lack of sub-population genetic structure. Genome-wide association results confirmed the complexity of the yield trait and the limited scope for searching individual QTLs with overwhelming effect on it. Some of our results concerning GBS procedures, SNP calling strategies, missing data imputation methods and statistical models for genomic selection can contribute to better design of genomic selection experiments for alfalfa and other crops with similar mating systems and commercial cultivar targets.

## Methods

### Reference population PV (Po Valley): composition and phenotyping

This reference population included elite germplasm from the Po Valley, Italy and was represented by a sample of 125 parental genotypes whose selection and phenotyping of their half-sib progenies for DM yield were described previously [1]. In brief, PV genotypes were chosen by stratified mass selection for DM yield over three harvests among 4480 densely planted genotypes grown in Lodi (Northern Italy) under field conditions in 2002 and 2003. The initial set of 4480 genotypes, whose origin and phenotypic variation are described in [23], represented well the germplasm from Northern Italy, including fixed amounts of randomly chosen genotypes from eleven farm landraces collected across the entire region and seven elite varieties. These populations had fall dormancy class 5 or 6, except for a few with dormancy 4 or 7. Half-sib progeny seed was obtained in summer 2005 by polycrossing the selected cloned genotypes in two large isolation cages, each including three complete crossing blocks with different genotype randomizations. Pollination in this and following intercrossing work was carried out by placing one micro-hive of bumblebees (*Bombus terrestris* L.) in each cage. We pooled the seed harvested from the six clones of each parent.

Half-sib progenies of the 125 genotypes were sown in jiffy pots and transplanted in November 2005 in a field experiment in Lodi that was designed as a randomized complete block with two replications. Each plot included 21 plants arranged in seven rows of three plants each, spacing plants at 0.12 m across and within rows, and using the two edge rows as border plants. Total DM yield was recorded across 12 harvests: five in 2006 and 2007 and two in spring 2008. The experiment received two irrigations of 30 mm each in 2006 and one irrigation of 40 mm in 2007, imposing a moderate level of summer drought stress that is consistent with the objective of selecting material widely adapted to Northern Italy [30]. On average, the experiment received 454 mm of water (rainfall plus irrigation) in the period March-October (when plant growth is substantial). DM yield values for genomic selection and genome-wide association studies were adjusted using BLUP computed from half-sib progeny mean values, as described in [32], i.e., by shrinking progeny main effects through multiplication by broad-sense heritability on a progeny mean basis (*h*_*B*_^*2*^) of the half-sib material estimated as:

$$ {h_B}^2={S_{hs}}^2/\left({S_{hs}}^2+{S_e}^2/r\right) $$

where *S*_*hs*_^*2*^ and *S*_*e*_^*2*^ are estimates of variance components for half-sib progeny and experiment error variation, and *r* is the number of experiment replications. One of the 125 parent genotypes was eliminated from genomic selection analyses, owing to poor quality (as number of reads) of its sequencing data.

### Reference population Me (Mediterranean): composition and phenotyping

This population represented elite germplasm adapted to Mediterranean-climate environments and included 154 parental genotypes that derived from two cycles of free intercrossing among three outstanding populations in a previous multi-environment study [24]. These populations, whose fall dormany class ranged from 7 to 10, were: (i) the drought-tolerant Sardinian landrace Mamuntanas; (ii) the salt-tolerant Moroccan landrace Erfoud 1; (iii) the Australian variety SARDI 10, widely adapted across moisture-favorable and drought-prone sites. The first intercrossing generation took place in 2009 and included 210 genotypes, 70 randomly chosen from each population. One seed per parent plant was harvested to establish the second intercrossing generation in 2010. One seed from each of 154 randomly-chosen parent plants on this generation was harvested, to establish the parent sample. Half-sib progeny seed of these 154 genotypes was obtained in 2011 and 2012 from three large isolation cages, each including three complete crossing blocks of randomized genotypes, pooling seed harvested over the nine clones of each parent.

Half-sib progenies of the 154 genotypes were sown in jiffy pots and then transplanted in mid-April 2012 in a field experiment in Lodi that was designed as an alpha lattice with three replications. Each plot included 36 plants arranged in nine rows of four plants each, spacing plants at 0.080 m across rows and 0.075 m within rows, and using the two edge rows as border plants. Phenotyping of Me focused on short-term DM yield in moisture-favorable, irrigated conditions. The experiment received 750 mm of water over the period March-October, assessing plot DM yields four times from June to October 2012. DM yield values were adjusted using BLUP as described for population PV.

### DNA isolation, GBS library construction and sequencing

DNA was isolated from fresh leaf tissues by the Wizard® Genomic DNA Purification Kit (Promega, A1125) and quantified with a Quant-iT PicoGreen dsDNA assay kit (Life Technologies, P7589). One library was constructed for each population, using the protocol by [19] with modifications. Briefly, 100 ng of each DNA was digested with ApeKI (NEB, R0643L) and then ligated to a unique barcoded adapter and a common adapter. Equal volume of the ligated product was pooled and cleaned up with QIAquick PCR purification kit (QIAGEN, 28104) for PCR amplification. In PCR, 50 ng template DNA was mixed with two primers and Taq polymerases in a 50 ul total volume. For the reference population Me, 5 nmoles each of the primers and NEB 2X Taq Master Mix (NEB Cat # M0270S) were included in the PCR reaction according to [19] original protocol. Amplification was carried out on a thermocycler for 18 cycles with 10 s of denaturation at 98 °C, followed by 30 s of annealing at 65 °C, and finally 30 s extension at 72 °C. For the reference population PV, we used a modified common adapter where “W” was changed to “A”, to reduce the number of target sites. The modifications in PCR included 25 nmoles of each primer instead of 5 nmoles, KAPA library amplification readymix (Kapa Biosystems Cat # KK2611) instead of NEB Taq Master Mix, and 10 cycles of reaction instead of 18. Each library was sequenced in two lanes on Illumina HiSeq 2000 at the Genomic Sequencing and Analysis Facility at the University of Texas at Austin, TX, USA.

### Genotype SNP calling

We used the UNEAK pipeline [33] for SNP discovery and genotype calling. The raw reads (100 bp, single end read) obtained from the sequencer were first quality-filtered and de-multiplexed. All reads beginning with the expected barcodes and cut site remnant were trimmed to 64 bp. Identical reads were grouped into one tag. Tags with 10 or more reads across all individuals were retained for pairwise alignment, which aimed to find tag pairs that differed by 1 bp. For each SNP marker, the reads distribution of the paired tags in each individual was used for SNP genotype calling. The three possible types of heterozygous of this autotetraploid species (i.e., Aaaa, AAaa and AAAa) were marked as diploid heterozygous (i.e. Aa), while the two homozygous were marked as diploid homozygous (i.e., AA or aa), according to [20]. One genotype of the PV population that generated a particularly low number of reads was discarded from all statistical analyses.

The SNP calling procedure was performed on each of the individual data sets (denoted PV_Sep and Me_Sep), and once on a joint data set obtained after collating the raw reads from the two sequencing runs. The latter calling procedure was used to create a consistent SNP naming across data sets in the absence of a reference genome. This joint data set was then split into two parts (denoted PV_Joint and Me_Joint) reflecting the two reference populations.

### Data filtering and imputation strategies

GBS can generate a great number of de-novo markers, but its information is typically limited by high number of missing values. The most-known and successful imputation algorithms were developed for species with a reference genome, which is missing in *M. sativa*, justifying our assessment of imputation algorithms as a function of their phenotype prediction accuracy. We considered four possible imputation algorithms, namely, MNI (Mean imputation), SVDI (Singular value decomposition imputation), RFI (Random forest imputation) and LHCI (Localized haplotype clustering imputation). For all algorithms, we imputed a M × N matrix of M individuals and N markers whose data points, defined in {0,1,2,NA}, represented the three possible genotypes and the missing value, respectively. MNI simply replaces each missing data point with the mean of the non-missing values for that marker, which are then discretized to the closer value in {0,1,2}. The algorithm was directly implemented as an R [34] function. SVDI operates a singular value decomposition on the genotype matrix to obtain a set of the k most significant eigen-vectors of the markers. These k eigen-vectors are used as the predictors for linear regression estimation of the missing data points, which are then discretized to the closer value in {0,1,2}. The algorithm was implemented using the R package “bcv” [35]. RFI uses random forest regression [36] to grow, for each missing data point, a set of random regression trees. We implemented RFI using the “MissForest” [37] R package, with the configuration ntree = 100, maxiter = 10, parallelize = ‘variables’. After the regression the imputed data were then discretized to the closer value in {0,1,2}. LHCI is implemented in the Beagle software [38] for use when a reference genome is available (since SNPs are imputed according to their physical order on chromosomes). We included it as a reference, repeating the analysis 20 times with different random reordering of imputed SNPs and verifying experimentally that SNP order had no influence on phenotype prediction models.

The four data sets (PV_Sep, Me_Sep, PV_Joint and Me_Joint) were filtered for increasing levels of allowed missing values, excluding SNPs whose missing rate over genotypes was greater than a fixed thresholds of 10, 20, 30, 40, 50 and 70 %. We estimated missing data according to each of the four imputation algorithms, and then filtered data to exclude markers with minor allele frequency < 2.5 %. Filtering and missing data estimations were performed independently for PV_Sep and Me_Sep data sets, and jointly for PV_Joint and Me_Joint (i.e., considering the joint matrix including 124 PV genotypes plus 154 Me genotypes). Our aim was verifying whether the greater information provided to imputation algorithms by joining the two data sets could result in greater accuracy of the genomic selection models.

We also created a COMMON data set that included only the SNPs that were consistently present in both data sets (hence, satisfying filtering criteria simultaneously for PV_Joint and Me_Joint data sets). COMMON was filtered over the same levels of missing data, and imputed with the four different algorithms.

### Population structure analysis

We verified the need for taking account of sub-populations and genetic structure in genomic selection and genome-wide association analyses by two methods applied separately to PV_Sep and Me_Sep data sets with 10 % SNP missing rate and RFI estimation of missing data. The first contemplated a Bayesian cluster analysis by the software STRUCTURE version 2.3 [39] using an admixture model with correlated allele frequencies, assessing the log likelihood values of posterior probability and the criterion proposed by [40] for optimal number of genotype groups across group numbers varying from 1 to 5. The analysis included six independent runs of 100,000 iterations preceded by a burn-in of 10,000 iterations. The second method explored the genetic relatedness between individuals through the analysis of the kinship matrix [41].

### Phenotype prediction for genomic selection

Different statistical models have been developed for genomic selection [15, 16]. We currently tested Ridge Regression BLUP, three Bayesian models, two Support Vector Regression models, and Random Forest Regression. The accuracy of predictions was assessed by Pearson’s correlation between predicted and observed phenotypes, splitting randomly 90 % genotypes to a training set and 10 % to a validation set. This cross-validation procedure was repeated 500 times, averaging the resulting accuracies.

Ridge regression BLUP (rrBLUP) assumes a linear mixed additive model where each marker is assigned an effect as a solution of the equation:

$$ y=\mu +G\kern0.5em u+\varepsilon $$

where *y* is the vector of observed phenotypes, *μ* is the mean of *y*, *G* is the genotype matrix (e.g., {0,1,2} for biallelic SNPs), *u* ~ *N (0, Iσ*^*2*^_*u*_*)* is the vector of marker effects, and *ε* is the vector of residuals. Solving with the standard ridge-regression method, the solution is:

$$ \widehat{u}=G\hbox{'}{\left(G\kern0.5em G\hbox{'}+\lambda \kern0.5em I\right)}^{-1}\left(y-\mu \right) $$

where *λ = σ*^*2*^_*e*_*/ σ*^*2*^_*u*_ is the ridge parameter, representing the ratio between residual and markers variance [42]. Given the vector of effects, it is then possible to predict phenotypes and estimate genetic breeding values. Ridge-regression BLUP analysis was performed through the R software package rrBLUP [43], estimating *λ* in a restricted maximum likelihood schema implemented by a spectral decomposition algorithm [44], and solving the resulting linear model.

Bayesian-based models assign prior densities to markers effects inducing different types of shrinkage. The solution is obtained by sampling from the resulting posterior density through a Gibbs sampling approach, as described by [45, 46]. We examined the phenotype prediction performances of three Bayesian prediction models, namely: (i) Bayes A [47]; (ii) Bayes B [48]; and (iii) the Bayesian Lasso [49]. Bayesian models were investigated by the R software package BGLR [50], using the following parameters: number of iterations = 5000; burn-in = 500; thinning = 5.

Support Vector Regression models are based on the computation of a linear regression function in a high dimensional feature space where the input data are mapped via a kernel function [29]. We considered two major kernel functions, namely, linear (SVR-lin) and gaussian (SVR-gau). We used the ε-insensitive regression present in the Weka framework [51], which ignores residuals smaller in absolute value than some constant (ε) and assigns a linear loss function for larger residuals. The regression was run using the following values: C = 1, ε = 0.1.

RFR is a combination of decision trees, each one generated from a subset of individuals selected by bootstrap [52]. RFR uses stochastic perturbation and averages the decision trees outputs to avoid over-fitting [53]. In this study the R package ‘RandomForest’ [54] was used with the following settings: number of variables tried at each split mtry = p/3, number of trees = 500 and minimum node size = 5.

We used SVR-lin and Ridge Regression BLUP consistently for all analyses, since these models tended to higher prediction accuracy than the other tested models. SVR-lin and SVR-gau displayed similar accuracies, but we preferred the former because of its faster computation time. For each reference population, genomic prediction using these models was explored for 48 data sets deriving from the combination of two SNP calling strategies, four imputation algorithms and six thresholds for missing data.

Genotypes of the COMMON data set were used for cross-population predictions based on SVR-lin and Ridge Regression BLUP, training the models on all genotypes of one population to predict the phenotypes of the other population. This analysis was performed for each of the six thresholds for missing data, using RFI. Phenotypes within each population were normalized to zero mean and unit variance prior to the analysis.

### Alignment to *M. truncatula* genome, and genome-wide association analysis

The Bowtie 2 tool [55] was used to query the consensus sequence of each tag pair containing a SNP against the *M. truncatula* reference genome Version 4.1 using the verysensitivelocal preset. SNP not aligning were placed in a fictitious chromosome N for visualization purposes. A genome-wide association analysis was conducted based on the EMMAX mixed model as described in [45] and implemented through the R package rrBLUP [44].

### Availability of supporting data

The data sets supporting the results of this article are available in the NCBI’s Sequence Read Archive (SRA) repository [Me population: http://www.ncbi.nlm.nih.gov/sra/SRX1421601, PV population: http://www.ncbi.nlm.nih.gov/sra/SRX1420586]. The information required to demultiplex the raw reads are provided in [Additional file 6] for PV data set and [Additional file 7] for Me data set.

## Additional files

Additional file 1: Figure S1. Frequency distribution of half-sib progeny dry matter yields for two data sets. Data for 124 parent genotypes of the Po Valley population (PV) and 154 parent genotypes of the Mediterranean population (Me). (PDF 18 kb) Additional file 2: Figure S2. Symmetric heatmap genetic kinship matrix between parent genotypes for two data sets. Data for 124 parent genotypes of the Po Valley population (PV) and 154 parent genotypes of the Mediterranean population (Me). Brighter color indicates greater genetic similarity. (PDF 99 kb) Additional file 3: Figure S3. Prediction accuracy for different genotype missing data imputation methods, SNP calling strategies and missing data thresholds. Results for four imputation methods (MNI, Mean imputation; SVDI, Singular value decomposition imputation; RFI, Random forest imputation; LHCI, Localized haplotype clustering imputation) applied to Po Valley (PV) and Mediterranean (Me) data sets subjected to separate SNP calling (PV_Sep and Me_Sep) or joint SNP calling (PV_Joint and Me_Joint), using Ridge Regression BLUP. (PDF 45 kb) Additional file 4:
**List of SNPs, with flanking sequences, detected in PV data set.** (ZIP 2612 kb) Additional file 5:
**List of SNPs, with flanking sequences, detected in Me data set.** (ZIP 2658 kb) Additional file 6:
**List of barcodes needed for sequence data demultiplexing for data set PV.** (TXT 13 kb) Additional file 7:
**List of barcodes, needed for sequence data demultiplexing for data set Me.** (TXT 23 kb)

## Abbreviations

GBS: genotyping-by-sequencing
LHCI: localized haplotype clustering imputation
MNI: mean imputation
SNP: single nucleotide polymorphism
QTL: quantitative trait loci
SVDI: singular value decomposition imputation
RFI: random forest imputation
rrBLUP: ridge regression best linear unbiased prediction
SVR-lin: support vector regression with linear kernel
SVR-gau: support vector regression with gaussian kernel
RFR: random forest regression
