# Supplementary figures and images for: Accuracy of genomic selection for alfalfa biomass yield in different reference populations
Source: BMC Genomics. 2015 Dec 1;16:1020. doi: 10.1186/s12864-015-2212-y (PMC4667460; doi:10.1186/s12864-015-2212-y)

Me

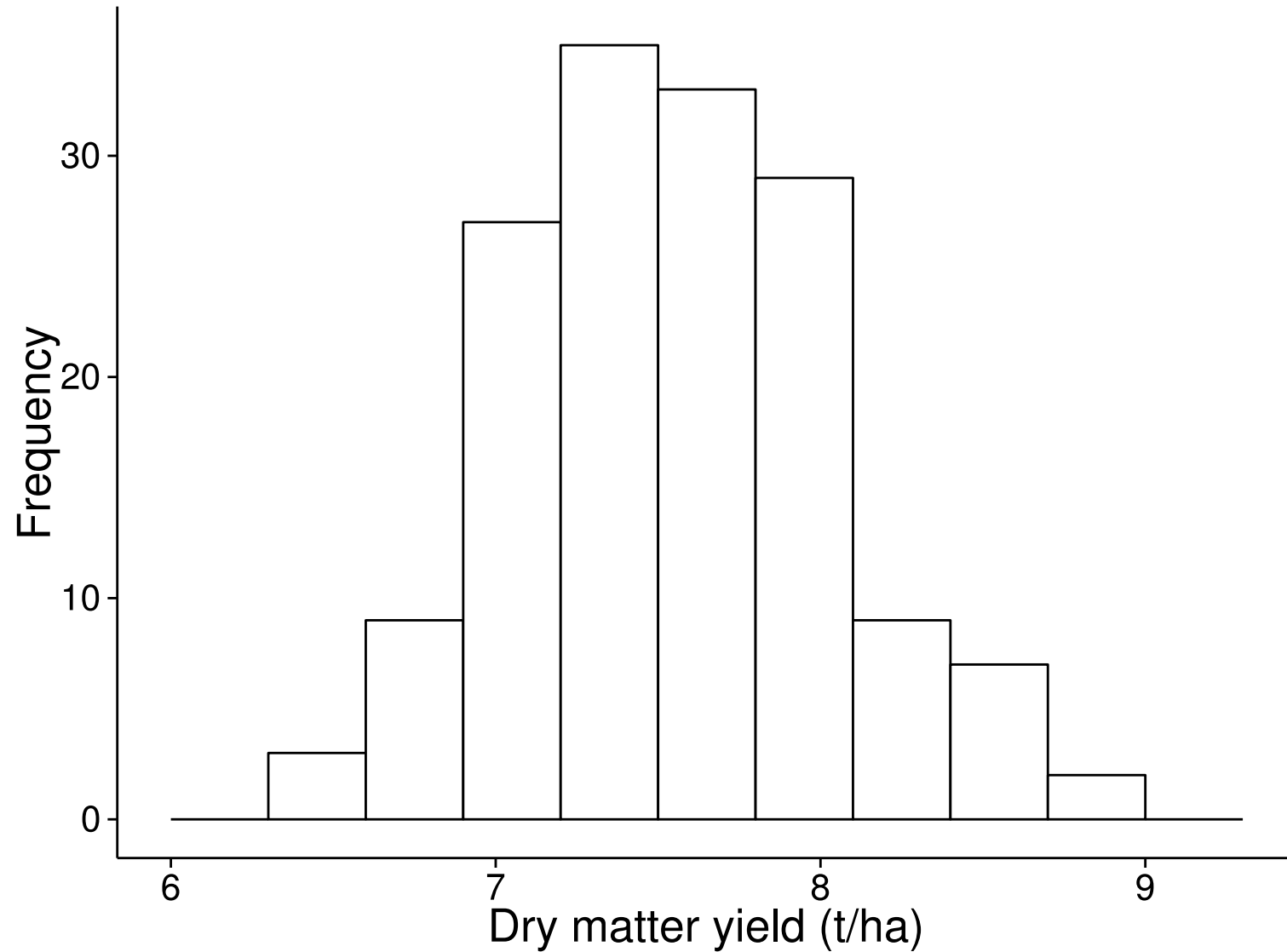

PV

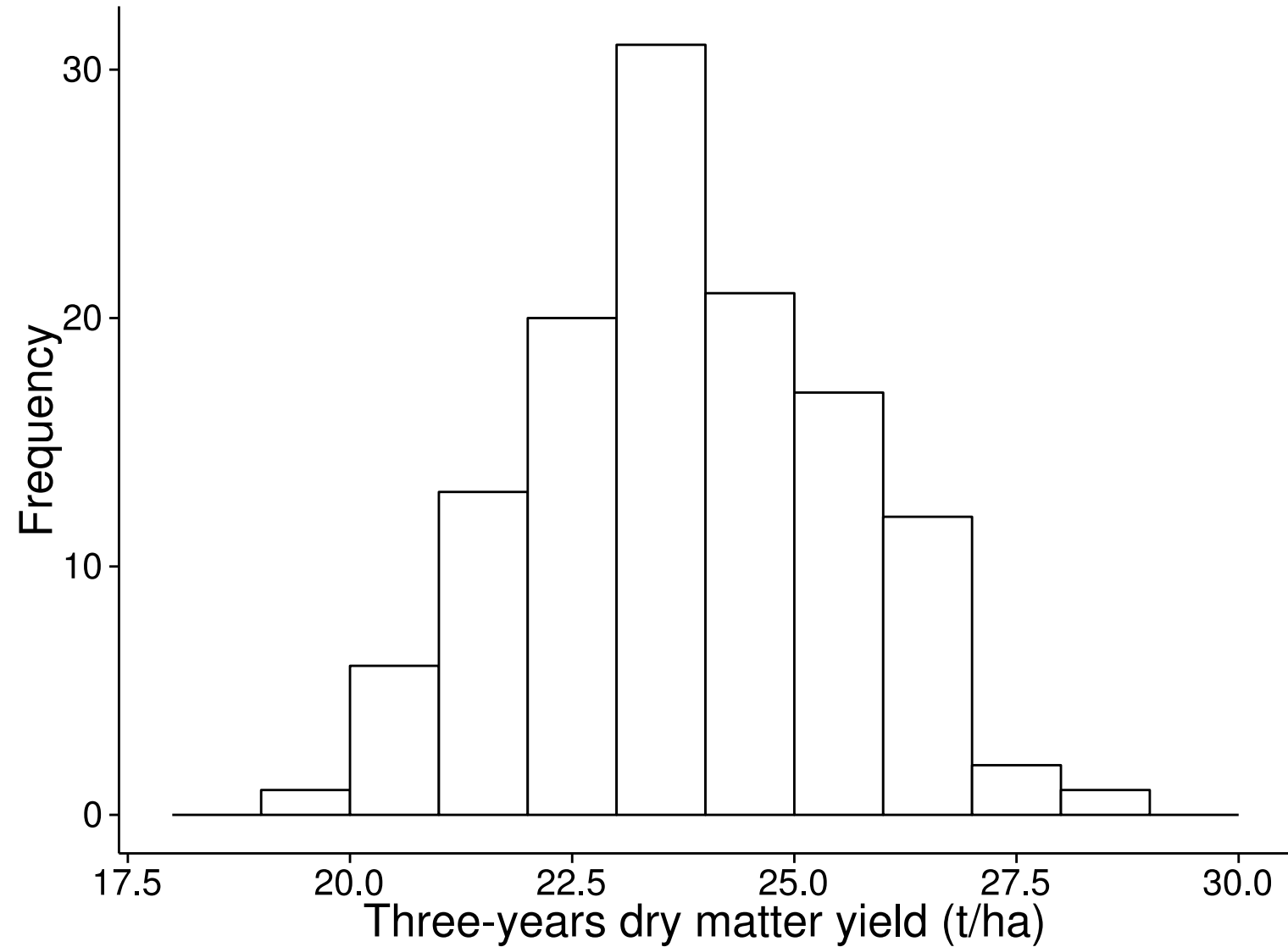

Supplement: Additional file 1: Figure S1. — Frequency distribution of half-sib progeny dry matter yields for two data sets. Data for 124 parent genotypes of the Po Valley population (PV) and 154 parent genotypes of the Mediterranean population (Me). (PDF 18 kb) [file 12864_2015_2212_MOESM1_ESM.pdf]

# PV\_sep

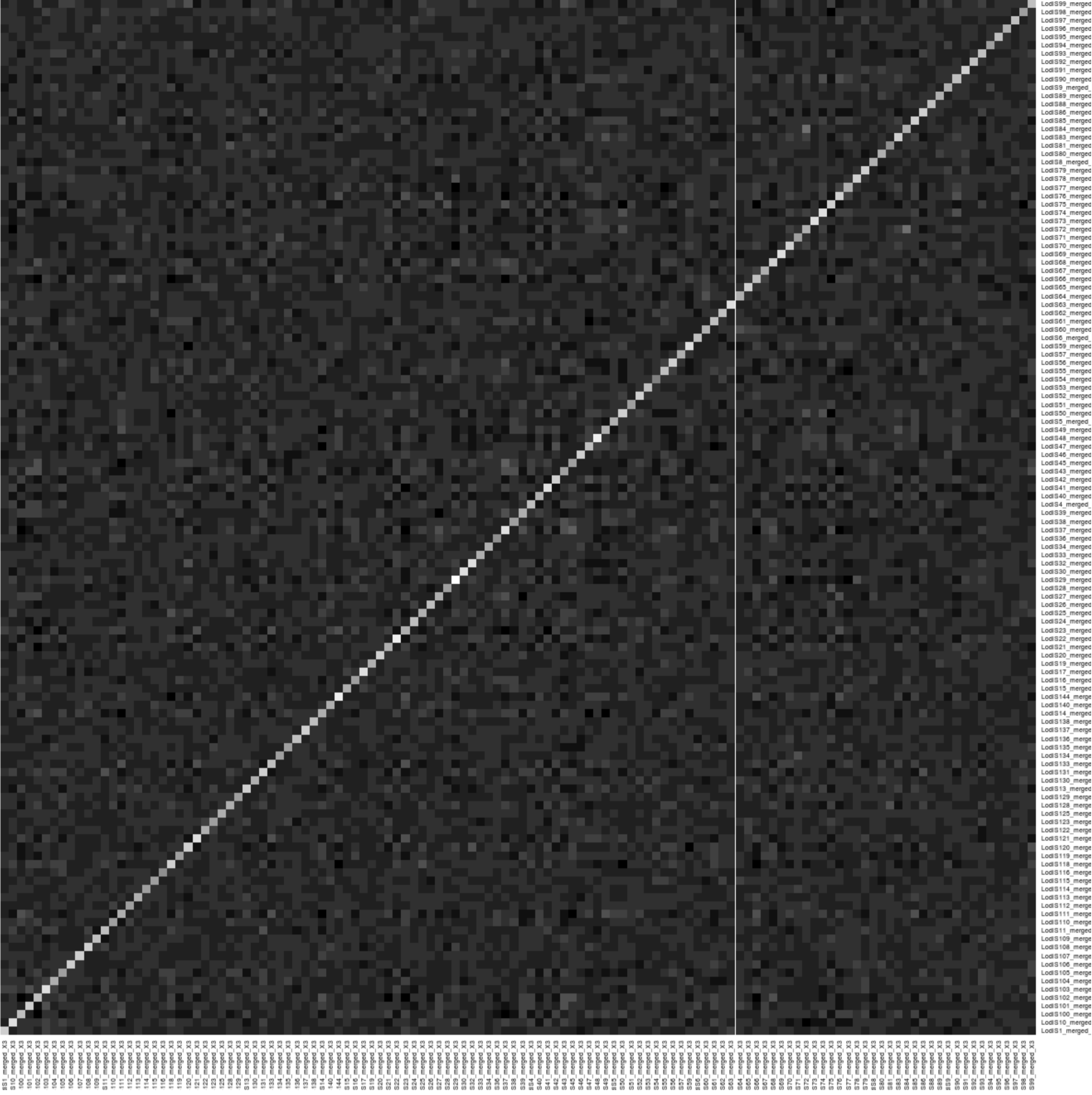

# Me\_sep

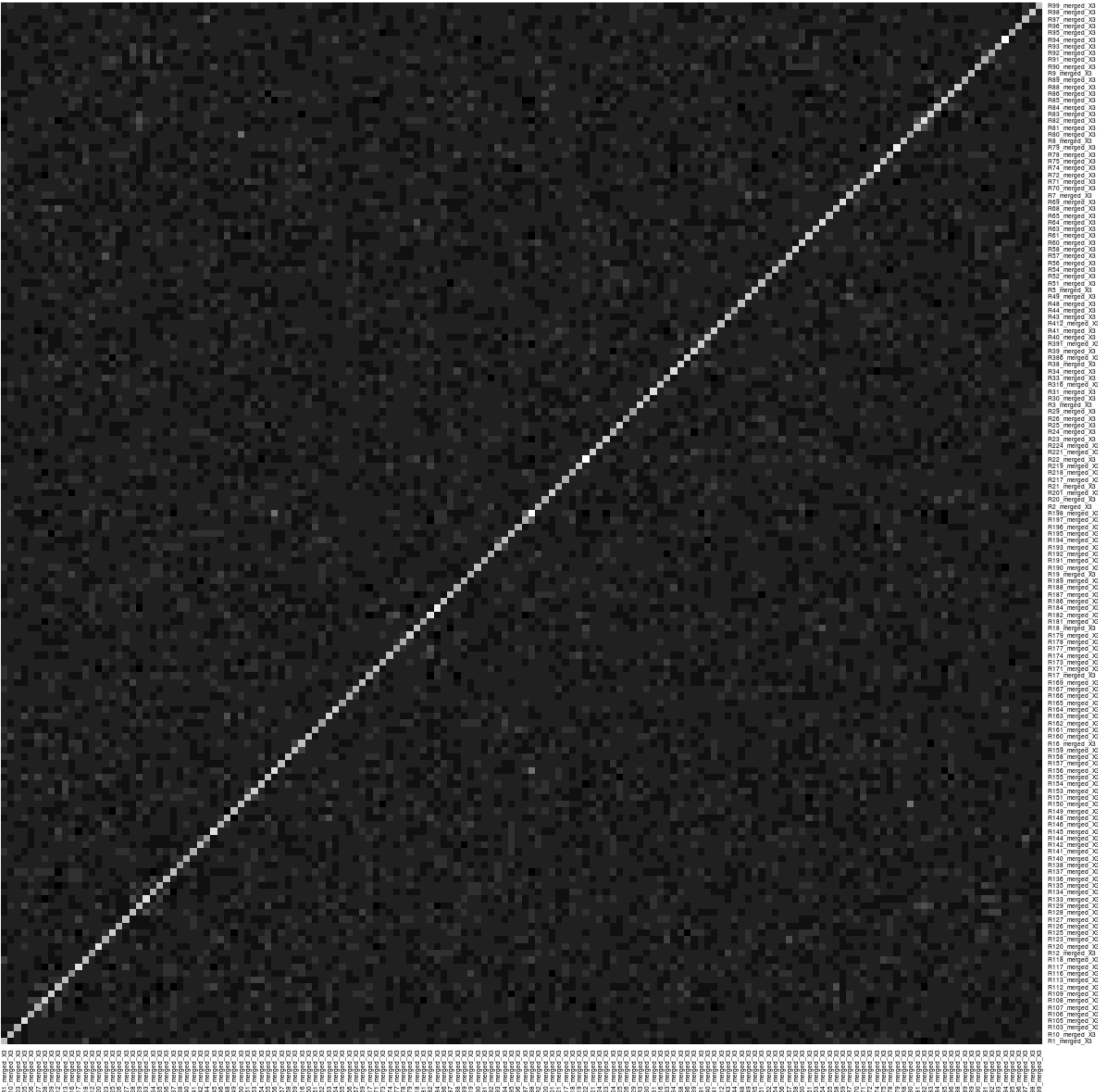

Supplement: Additional file 2: Figure S2. — Symmetric heatmap genetic kinship matrix between parent genotypes for two data sets. Data for 124 parent genotypes of the Po Valley population (PV) and 154 parent genotypes of the Mediterranean population (Me). Brighter color indicates greater genetic similarity. (PDF 99 kb) [file 12864_2015_2212_MOESM2_ESM.pdf]

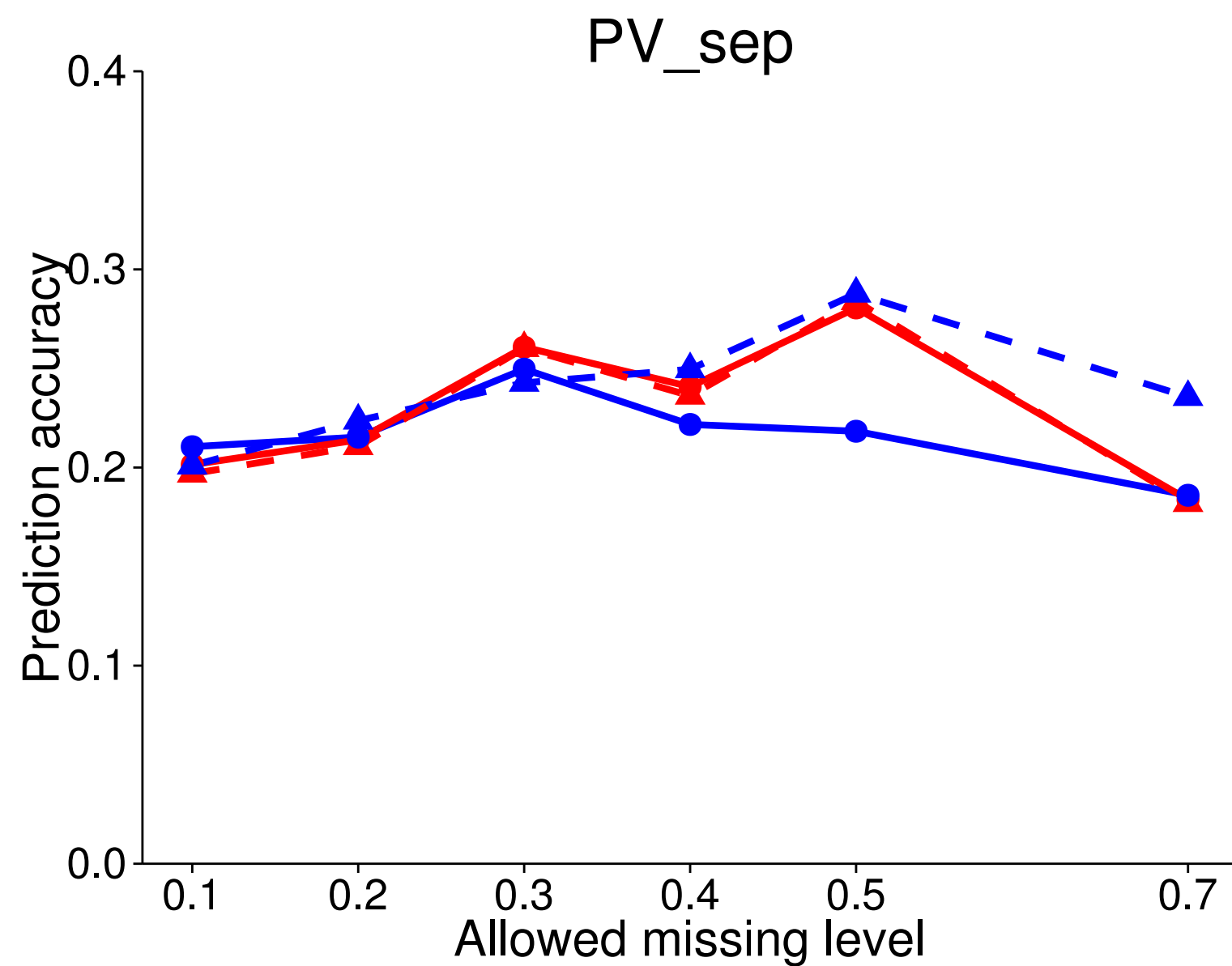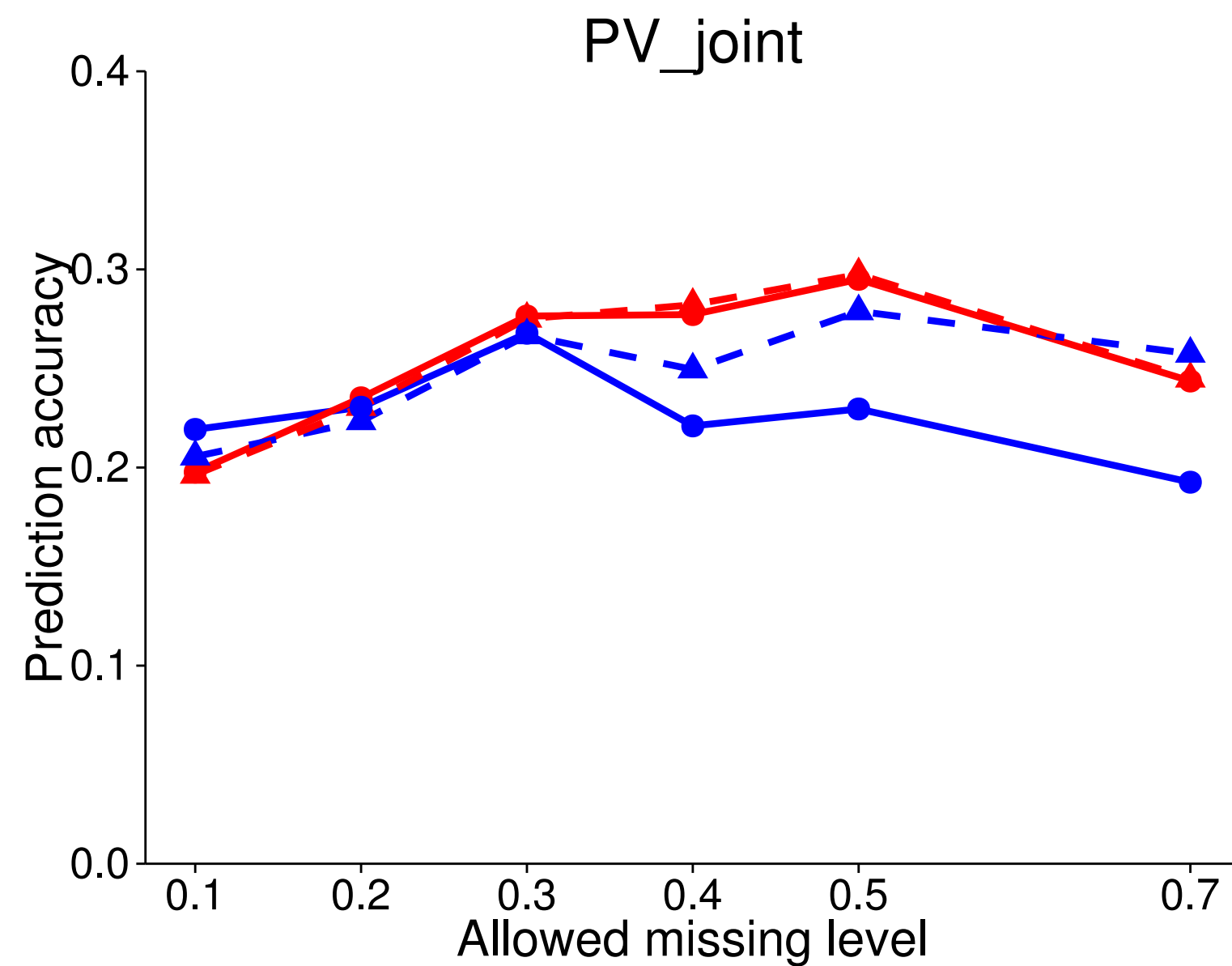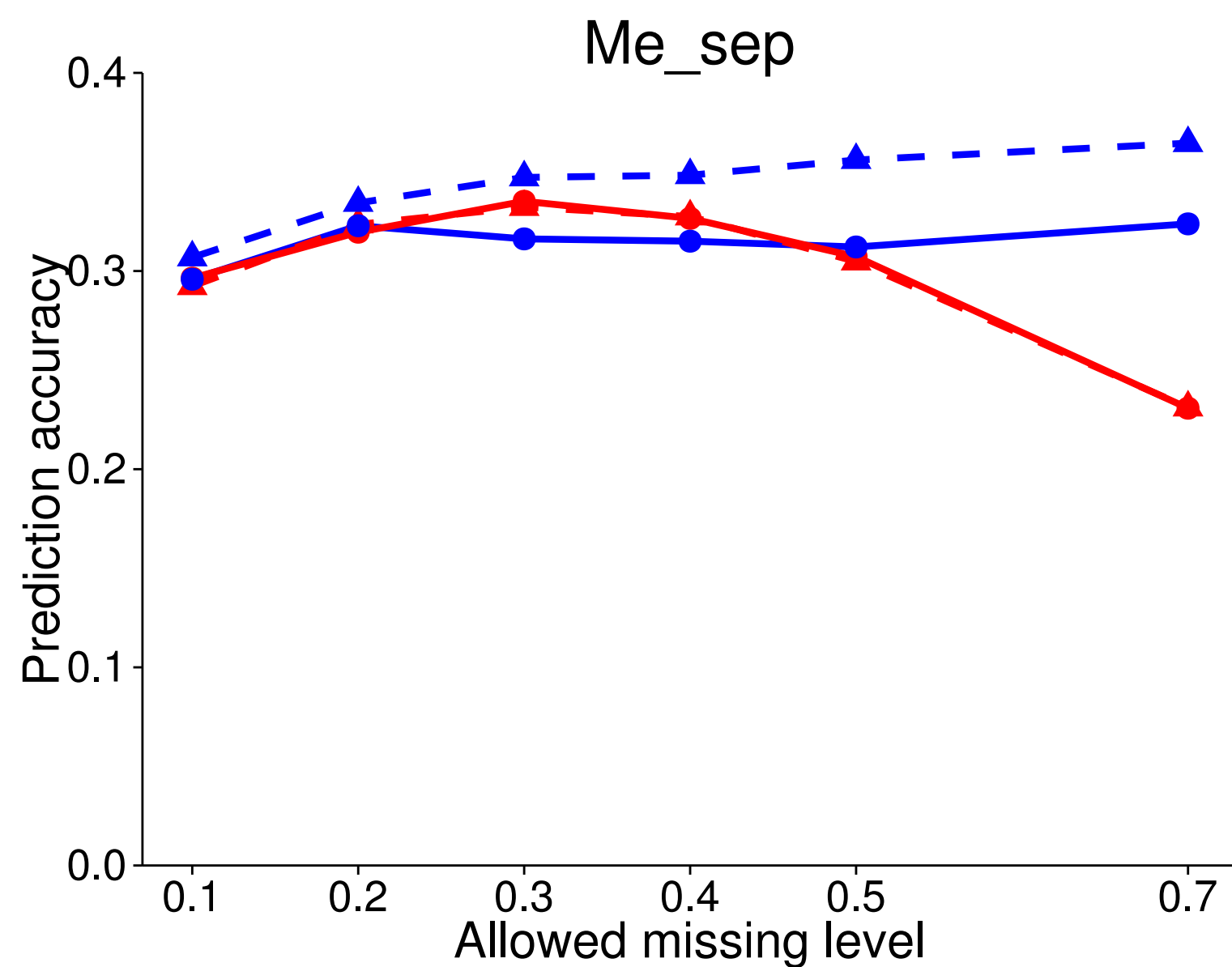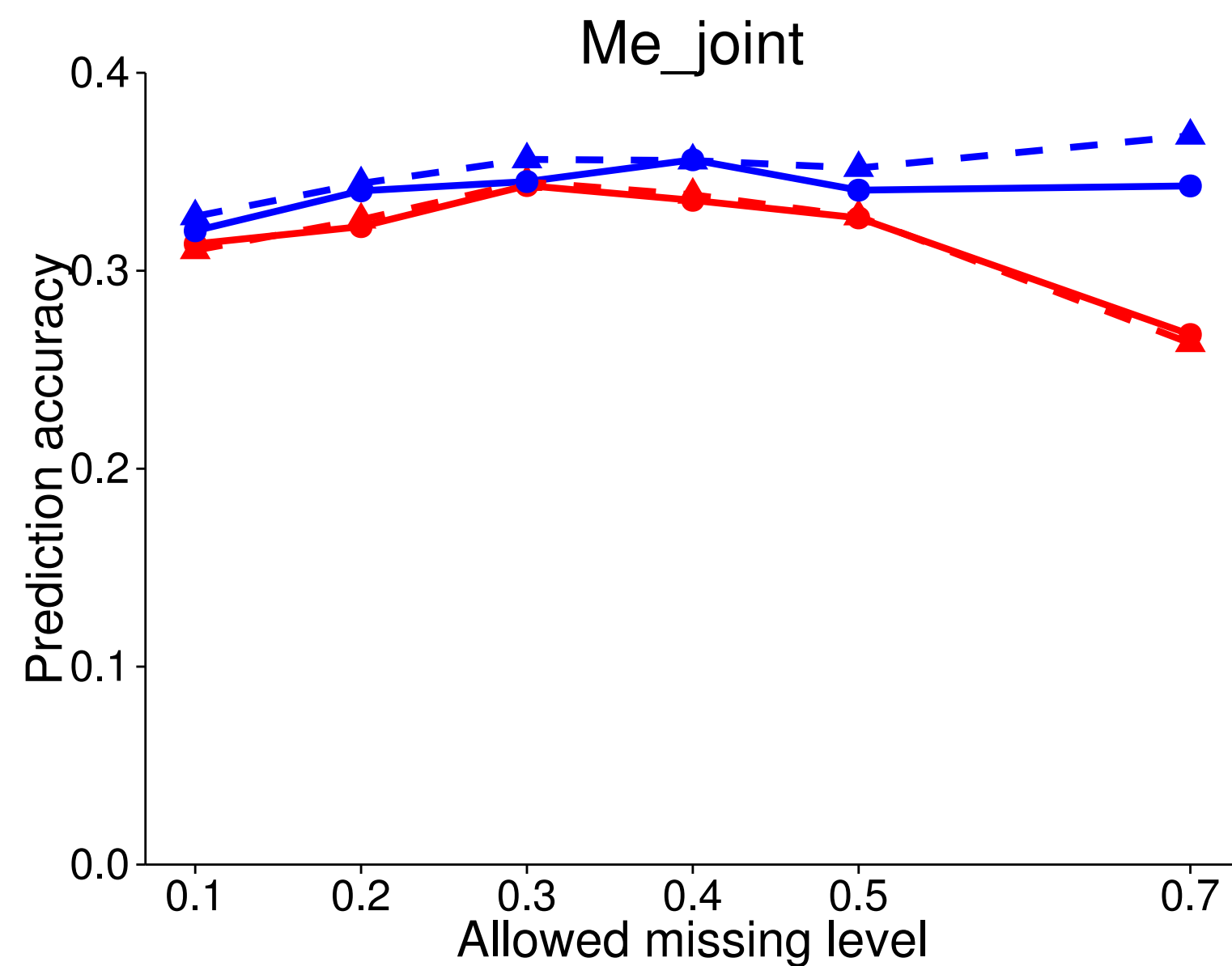

● LHCI ● MNI ▲ RFI ▲ SVDI

Supplement: Additional file 3: Figure S3. — Prediction accuracy for different genotype missing data imputation methods, SNP calling strategies and missing data thresholds. Results for four imputation methods (MNI, Mean imputation; SVDI, Singular value decomposition imputation; RFI, Random forest imputation; LHCI, Localized haplotype clustering imputation) applied to Po Valley (PV) and Mediterranean (Me) data sets subjected to separate SNP calling (PV_Sep and Me_Sep) or joint SNP calling (PV_Joint and Me_Joint), using Ridge Regression BLUP. (PDF 45 kb) [file 12864_2015_2212_MOESM3_ESM.pdf]
